# Glucose-6-phosphate dehydrogenase (G6PD) deficiency in Ethiopia: absence of common African and Mediterranean allelic variants in a nationwide study

**DOI:** 10.1186/s12936-018-2538-4

**Published:** 2018-10-26

**Authors:** Ashenafi Assefa, Ahmed Ali, Wakgari Deressa, Wendimagegn Tsegaye, Getachew Abebe, Heven Sime, Amha Kebede, Daddi Jima, Moges Kassa, Tesfay Abreha, Hiwot Teka, Hiwot Solomon, Joseph Malone, Ya Ping Shi, Zhiyong Zhou, Richard Reithinger, Jimee Hwang

**Affiliations:** 1 Ethiopian Public Health Institute, Addis Ababa, Ethiopia; 2 Addis Ababa University, Addis Ababa, Ethiopia; 3 Columbia University ICAP, Addis Ababa, Ethiopia; 4 US President’s Malaria Initiative, United States Agency for International Development, Addis Ababa, Ethiopia; 5 Ethiopian Federal Ministry of Health, Addis Ababa, Ethiopia; 6 US President’s Malaria Initiative, Malaria Branch, Division of Parasitic Diseases and Malaria, Centers for Disease Control and Prevention, Atlanta, GA USA; 7 Malaria Branch, Division of Parasitic Diseases and Malaria, Centers for Disease Control and Prevention, Atlanta, GA USA; 8 RTI International, Washington, DC USA; 9 Global Health Group, University of California San Francisco, San Francisco, CA USA

**Keywords:** Ethiopia, Malaria, G6PD deficiency, Primaquine

## Abstract

**Background:**

Building on the declining trend of malaria in Ethiopia, the Federal Ministry of Health aims to eliminate malaria by 2030. As *Plasmodium falciparum* and *Plasmodium vivax* are co-endemic in Ethiopia, the use of primaquine is indicated for both transmission interruption and radical cure, respectively. However, the limited knowledge of the local prevalence of glucose-6-phosphate dehydrogenase (G6PD) deficiency and its associated variants has hindered the use of primaquine.

**Methods:**

Some 11,138 dried blood spot (DBS) samples were collected in 2011 as part of a national, household Malaria Indicator Survey, a multi-stage nationally representative survey of all malaria-endemic areas of Ethiopia. A randomly selected sub-set of 1414 DBS samples was successfully genotyped by polymerase chain reaction–restriction fragment length polymorphism (PCR–RFLP) technique. Considering the geographical position and ethnic mix of the country, three common variants: G6PD*A (A376G), G6PD*A− (G202A) and Mediterranean (C563T) were investigated.

**Results:**

Of the 1998 randomly selected individuals, 1429 (71.5%) DBS samples were genotyped and merged to the database, of which 53.5% were from females. G6PD*A (A376G) was the only genotype detected. No sample was positive for either G6PD*A− (G202A) or Mediterranean (C563T) variants. The prevalence of G6PD*A (A376G) was 8.9% [95% confidence interval (CI) 6.7–11.2] ranging from 12.2% in the Southern Nations, Nationalities and Peoples’ (95% CI 5.7–18.7) to none in Dire Dawa/Harari Region.

**Conclusion:**

The common G6PD*A− (G202A) or Mediterranean (C563T) variants were not observed in this nationwide study. The observed G6PD*A (A376G) mutation has little or no clinical significance. These findings supported the adoption of primaquine for *P. falciparum* transmission interruption and radical cure of *P. vivax* in Ethiopia. As the presence of other clinically important, less common variants cannot be ruled out, the implementation of radical cure will be accompanied by active haematological and adverse events monitoring in Ethiopia.

## Background

In Ethiopia, malaria is caused by both *Plasmodium falciparum* (63.7% of all confirmed cases) and *Plasmodium vivax* (36.3%) [1]. Due to the scale-up of key malaria interventions in the last decade, several districts are now in the pre-elimination phase [2]. A drug capable of interrupting *P. falciparum* malaria transmission and radical cure of *P. vivax* has the potential to accelerate malaria control and elimination efforts. Primaquine, an 8-aminoquinoline, is currently the only generally available drug for these indications in malaria endemic countries. The widespread use of primaquine has been limited due to its potential to induce haemolytic anaemia in glucose-6-phosphate dehydrogenase (G6PD)-deficient individuals. Haemolytic anaemia in G6PD-deficient individuals can range from mild to life-threatening conditions [3–5]. Another 8-aminoquinoline, tafenoquine, completed Phase 2 and 3 trials and recently was approved by the US Food and Drug Agency for radical cure of *P. vivax* infections [6].

G6PD deficiency is an X-linked genetic disorder and it is estimated to affect more than 400 million people worldwide [4, 7]. It is one of the most prevalent polymorphisms and enzymopathies in humans and is found more commonly in malaria-endemic areas [4, 7]. In Ethiopia, there is limited information about the occurrence and distribution of G6PD deficiency [7]. There has been no nationally representative study on its prevalence and distribution conducted to date. Sporadic studies note the absence of G6PD deficiency in highland areas of the country, while in lowland areas G6PD deficiency prevalence ranged from 1.4 to 14.3% in the southwest (Nuer and Anuak) and northeast (Aregobas in Afar) regions concentrated in certain ethnic groups [8–11].

Because of substantial progress made in malaria control, Ethiopia’s vision is to be malaria-free by 2030 [2, 12–14]. Determining the G6PD prevalence, distribution and variants can inform the Federal Ministry of Health’s decision to adopt new policies to implement and scale-up the use of primaquine for both *P. falciparum* transmission reduction and *P. vivax* radical cure without prior G6PD testing, which will be crucial in achieving their vision. This study aimed to evaluate the genotypic prevalence of common G6PD deficiency allelic types in the general population residing in malaria-endemic areas of Ethiopia.

## Methods

### Study design and analysis

Dried blood spot (DBS) samples collected during the national Malaria Indicator Survey in 2011 (MIS 2011) were used for the current study. The MIS 2011 was a cross-sectional, multi-stage, representative household survey that produced national and sub-national estimates for malaria-endemic and malaria prone areas of Ethiopia [15]. Using a two-stage cluster sampling methodology, 25 households were selected from each enumeration areas (average size 175 households) by simple random sampling. In each selected household, blood samples were collected for all children under 5 years of age and all family members in every fourth household [15].

A total of 11,138 DBS samples were collected and stored at − 20 °C as part of the MIS 2011 [15]. Representing the geographic regions that had been sampled during the survey, 1998 individuals were randomly selected. The MIS 2011 sampling protocol used a non-proportional allocation of samples proportional to population size. A sample size of 1998 was selected assuming a sample proportion of 0.03 to produce a two-sided 95% confidence interval with a width equal to 0.015 [16]. To ensure the representativeness of the samples, sampling weights were calculated by performing a weighting class adjustment to force weights from those tested for G6PD sub-sampling to equal the total weight in the full sample. Adjustments were made by province, altitude, and age group. Clustering at the village level was also accounted. PASS 11 (NCSS, LLC. Kaysville, Utah, USA) and SAS 9.4 (SAS Institute Inc, Cary, NC, USA) were used in sample size determination and data analysis, respectively.

### Genotyping: PCR–RFLP

Based on a review of the literature, the country’s geographic proximity to the Arabian peninsula and ethnic mix, the following three G6PD mutations common in Africa and the Mediterranean Regions were selected for genotyping: G6PD*A (A376G), G6PD*A− (G202A) and Mediterranean (C563T) [4, 7, 17].

A polymerase chain reaction-restriction fragment length polymorphism (PCR–RFLP) technique was used for genotyping G6PD based on previous publications [19, 20] modifying the annealing temperature to reduce nonspecific amplification. Briefly, DNA was extracted from DBS samples using the Qiagen DNA mini-kit (Qiagen, Germantown, USA). PCR was used for the amplification of three specific regions. Genomic DNA was first amplified using primers for G6PD*A (A376G), 5′-CCCAGGCCACCCCAGAGGAGA-3′ (forward) and 5′-CGGCCCCGGACACGCTCATAG-3′ (reverse) and all samples positive for G6PD*A (A376G) were then subjected to PCR amplification using primers for G6PD*A− (G202A), 5′-CACCACTGCCCCTGTGACCT-3′ (forward) and 5′-GGCCCTGACACCACCCACCTT-3′ (reverse). All DNA extracts were also amplified for Mediterranean (C563T) type mutation using primers 5′-AGCTCTGATCCTCACTCCCC-3′ (forward) and 5′-GGCCAGGTGAGGCTCCTGAGTA-3′ (reverse). The following PCR conditions were used for 376 and 202 mutations; initial degeneration one cycle for 5 min at 94 °C, then 32 cycles for degeneration of 45 s at 94 °C, annealing of 30 s at 64 °C, extension of 45 s at 72 °C, and final extension at 72 °C for 1 min. Similar PCR conditions were used for Mediterranean (C563T) mutation with the following modifications; initial degeneration one cycle for 5 min at 94 °C, then 32 cycles for degeneration of 1 min at 94 °C, annealing of 1 min at 57 °C, extension of 1 min at 72 °C and final extension at 72 °C for 1 min.

**Fig. 1:**
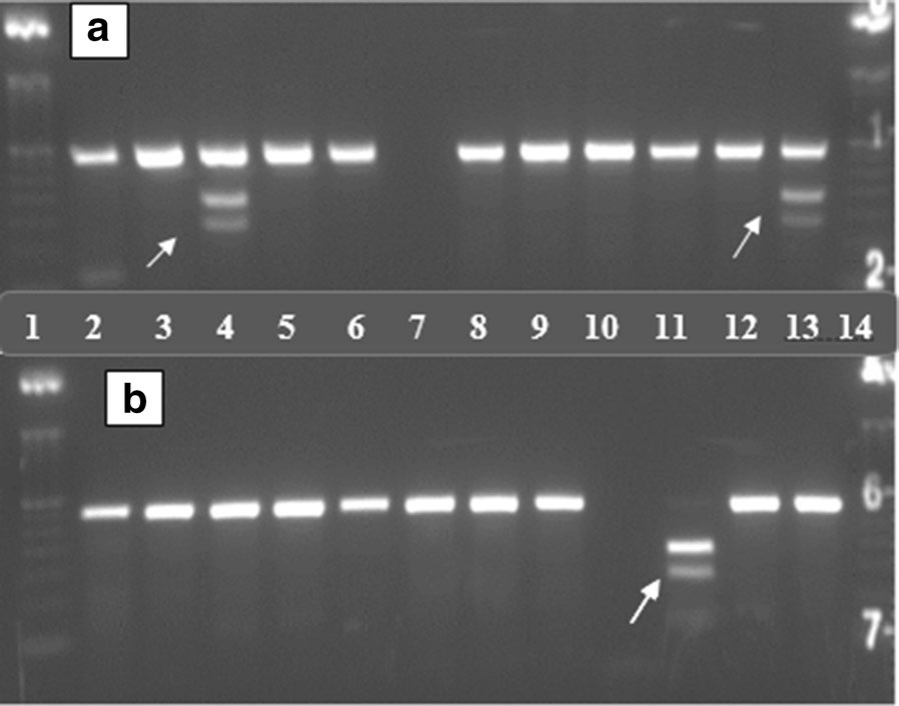
Picture of gel electrophoresis result showing G6PD*A (A376G) mutation. Lane 1 indicates a molecular ladder of 50 bp. Arrows indicate the site for mutation. **a** Lane A4 and A13 show partial digestion, cut size 308 (undigested), 183 and 125 bp. **b** Lane B11 show full digestion, cut size 183 and 125 bp

Three restriction endonuclease enzymes, FokI (G6PD*A (A376G)), NlaIII (G6PD*A− (G202A)) and MboII (Mediterranean (C563T)) (New England Biolabs Ltd, Ipswich, MA), were used for the digestion of the amplified products (Table 1). Samples were incubated with the restriction enzymes for 1 h at 37 °C, followed by inactivation at 65 °C for 25 min [19–22]. Final PCR–RFLP products were separated using 2% agarose gel-electrophoresis and visualized under UV illumination (Fig. 1).

**Table 1:** Types of G6PD variants screened by RFLP and expected size of fragments in base pair

Mutation	Variants	Restriction enzymes	Size	Fragment Size		References
				Wild type	Mutant	
376 A→G	G6PD B→G6PD A	FokI	308	308	125, 183	[19]
202 G→A	A^**−**^	NlaIII	211	211	81, 130	[19]
563 C→T	Mediterranean	MboII	285	33, 252	33, 98, 154	[18]

Known blood samples archived at CDC, collected from a cross-sectional survey conducted in Kenya and a malaria in pregnancy project conducted in Malawi served as positive controls for G6PD*A (A376G) and G6PD*A− (G202A) (African type mutations). However, there were no positive controls used for Mediterranean (C563T) type mutations. Results with faint and intermediate bands were repeated by using the concentrated DNA samples to achieve more distinct test results. The testing was conducted at the Malaria and other Parasitic Diseases Research Team at the Ethiopian Public Health Institute; a sub-set of samples (10%) was genotyped at CDC Atlanta for quality assurance testing and training.

## Results

Of the 1998 randomly selected samples, only 1641 (82.1%) DBS were found. DNA was extracted and successfully amplified from 1548 samples (94.3%). An additional 119 samples were not able to be merged to the original database, resulting in 1429 merged genotype outcomes and 1414 with valid genotype results (15 samples with unsatisfactory gel results after repeated attempts). Of all the samples analysed and merged to the database, 53.5% (764/1429) were from females.

**Fig. 2:**
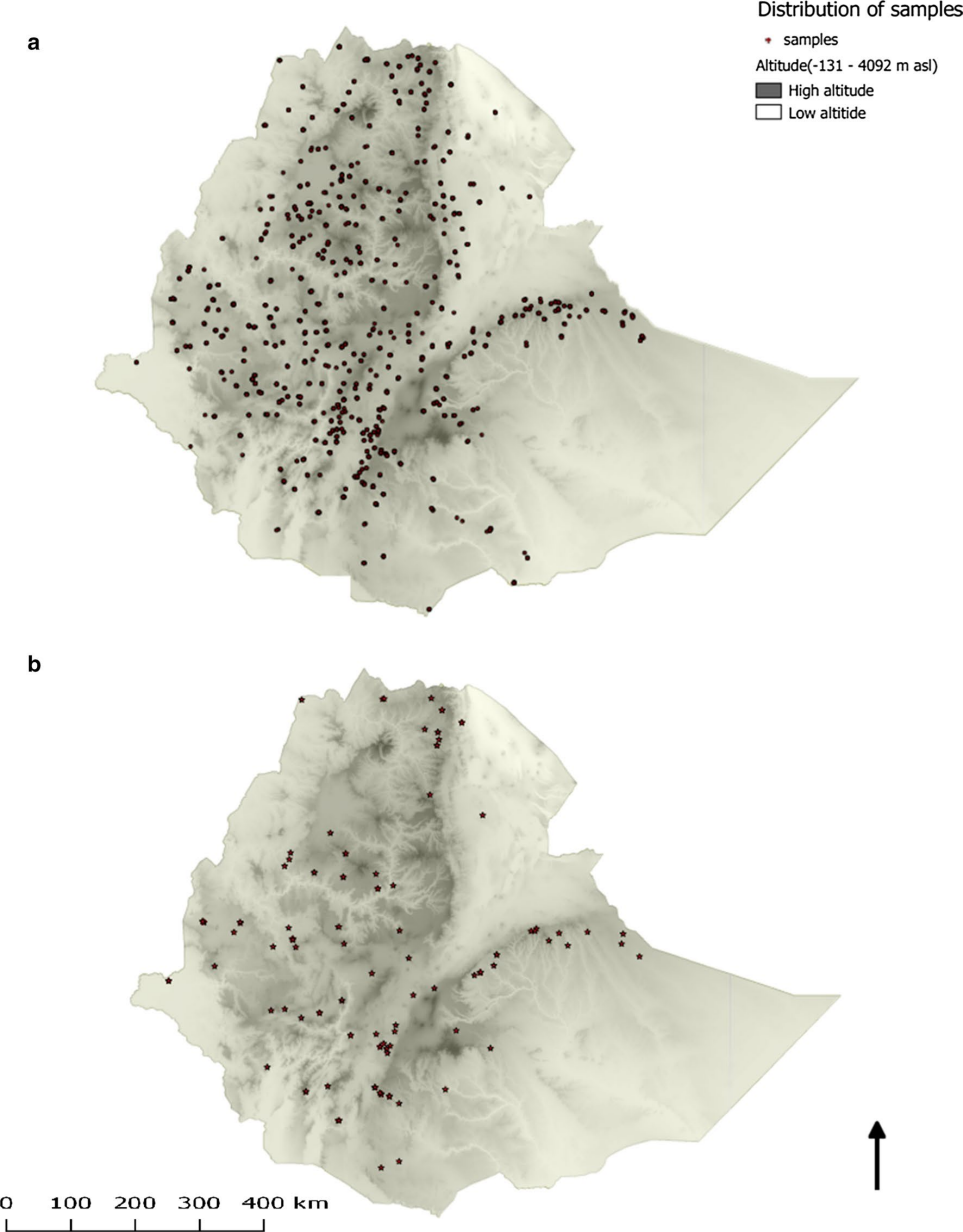
Spatial distribution of study samples in Ethiopia. **a** Distribution of samples selected for G6PD genotyping (n = 1947; 51 samples missing GPS coordinates). **b** Spatial distribution of G6PD*A mutations (n = 130). Darker color depicts higher altitude and lighter color lower altitude. Areas above 2500 m were considered unsuitable for malaria transmission and were not included in the sampling frame. Each dot represents a household where a DBS was collected

Figure 2a shows the spatial distribution of the samples selected. Similar to the overall MIS 2011 estimates [15], the average age of the study participants was 14.3 (range 1–89) years. Although samples were randomly selected without considering signs and symptoms of malaria or malaria testing results, 19 samples were positive by malaria rapid diagnostic tests (15 *P. falciparum* and 4 *P. vivax*).

Table 2 shows the weighted genotyping results by region. The study showed that the G6PD*A (A376G) mutation was the only mutation found in 8.9% (95% confidence interval (CI) 6.7–11.2) of the analysed samples. Of the G6PD*A (A376G) mutations, 57.8% (75/130) was observed in females. The G6PD*A− (G202A) and Mediterranean (C563T) mutations were not detected. No spatial clustering was observed for the G6PD*A (A376G) mutation (Fig. 2b) which was similar to the spatial distribution of all samples selected (Fig. 2a).

**Table 2:** Number of DBS samples analysed and prevalence of G6PD*A (A376G) mutants by region

Region	No. of samples extracted	No. of samples amplified	No. of samples merged for analysis	No. G6PD*A (A376G) mutants observed	Weighted prevalence (%) of G6PD*A (A376G) mutation (95% CI)
Afar and Somali	152	128 (84.2%)	118 (77.6%)	7	7.7 (0.5–14.9)
Amhara	302	262 (86.8%)	229 (75.8%)	15	7.5 (3.0–12.0)
Benishangul-Gumuz and Gambella	118	117 (99.2%)	109 (92.4%)	8	5.3 (0.7–10.0)
Dire Dawa and Harari	11	11 (100%)	10 (90.9%)	0	0
Oromia	640	633 (98.9%)	576 (90.0%)	58	8.0 (5.0–10.9)
Southern Nations, Nationalities, and Peoples’	277	256 (92.4%)	240 (86.6%)	28	12.2 (5.7–18.7)
Tigray	141	141 (100%)	132 (93.6%)	14	12.0 (4.4–19.5)
Total	1641	1548	1414	130	8.9 (6.7–11.2)

No G6PD*A− (G202A) and Mediterranean (C563T) variants were observed

Although not statistically significant, there was regional variation in the prevalence of the G6PD*A (A376G) mutation: the highest prevalence was observed in Southern Nations, Nationalities, and Peoples’ and Tigray Regions (12.2%; 95% CI 5.7–18.7 and 12.0%; 95% CI 4.4–19.5, respectively), compared to Dire Dawa and Harari where no G6PD mutations were observed (P = 0.278). The prevalence of G6PD*A (A376G) was 9.2% (95% CI 6.7–11.8) in areas with altitude less than 2000 m, and 7.9% (95% CI 2.4–13.4) in areas with altitude between 2000 and 2500 m above sea level (P = 0.403).

## Discussion

In this nationally representative study, 8.9% of the samples were noted to carry the G6PD*A (A376G) variant; the clinically relevant variants of G6PD*A− (G202A) and Mediterranean (C563T) were not observed. Although the more clinically important type G6PD*A− (G202A) is found in a sub-set of individuals with the G6PD*A (A376G) variant in other studies [5, 18, 23], none of the G6PD*A (A376G) mutant genes were noted to carry the G6PD*A− (G202A) mutation in this study. The G6PD*A (A376G) mutation observed in the study is a common variant, resulting in close to normal (~ 85%) enzyme activity of a non-deficient person, without significant clinical manifestations of G6PD-related haemolysis or appearing to confer resistance to malaria [5, 21, 24–27]. The analysis of Mediterranean (C563T) deficiency was included in this study because of the severity of the variant, the proximity of Ethiopia to the Mediterranean and Middle East Regions, and the ethnic mix observed in country [28, 29]; however, this severe variant was not observed.

The results are consistent with prior evidence of low G6PD deficiency prevalence in Ethiopia [7, 9–11, 25]. For example, the absence of G6PD*A− (G202A) and Mediterranean (C563T) mutations was recently reported from blood samples obtained from malaria patients in Southwestern Ethiopia, where 23% of the individuals were noted to carry the G6PD*A (A376G) mutation [22].

There are several limitations to this evaluation. First, the large sample loss (29.2%) mainly due to not being able to locate the corresponding DBS, is a major limitation, although there is likely little or no systematic bias introduced in this random loss. Second, the current study only tested for three common variants of the hundreds of known G6PD variants [25]. The current study cannot definitively conclude that the overall prevalence of clinically relevant G6PD-deficient variants is low or it excluded all clinically relevant variants found in Ethiopians (e.g., G6PD Rehovot [30] especially from regions with very few samples. Recent sequencing of samples from Southwestern Ethiopia have identified uncommon mutations, including a new mutation not previously reported [22], although their phenotypes were not documented [31]. Third, the current study only reports the genotypic prevalence of the variants which does not imply phenotypic prevalence. Tsegaye et al. [11] using the Carestart™ G6PD RDT reported high phenotypic prevalence of G6PD deficiency in Gambela Region (Anuak and Nuer ethnic groups), thus, further studies are needed to identify the associated variants and haemolytic profile.

## Conclusion

No common, G6PD deficiency variants (G6PD *A−) and Mediterranean (C563T) were found in this nationwide study. Following a review of the data from prior studies and the current study, the Federal Ministry of Health has adopted the addition of single low dose primaquine for transmission reduction of *P. falciparum*. In addition, they are adopting a policy for the first time in decades of primaquine use for radical cure of *P. vivax* infections without G6PD testing, starting in low transmission highland districts with directly observed therapy and close supervision [32]. Ethiopia, especially the highlands, may represent a lower risk location for the use of primaquine in the radical cure for *P. vivax* malaria without G6PD testing. The safety of implementing this policy will be evaluated through active adverse events and haematological monitoring using a standardized approach with common endpoints [33]. The risks and benefits of primaquine radical cure without G6PD testing will need to be further assessed in Ethiopia as the *P. vivax* case management landscape evolves to incorporate developing regions in the lowlands, new evidence around higher dose and shorter course of primaquine and as new point-of-care, quantitative G6PD diagnostics and tafenoquine become available [6, 33–35].

## Abbreviations

CI: confidence interval
DBS: dried blood spot
G6PD: glucose-6-phosphate dehydrogenase
MIS: malaria indicator survey
PCR: polymerase chain reaction
RFLP: restriction fragment length polymorphism
SNNPR: Southern Nations and Nationalities People’s Region
